# The Potential of α-Mangostin from *Garcinia mangostana* as an Effective Antimicrobial Agent—A Systematic Review and Meta-Analysis

**DOI:** 10.3390/antibiotics11060717

**Published:** 2022-05-26

**Authors:** Omer Sheriff Sultan, Haresh Kumar Kantilal, Suan Phaik Khoo, Amalraj Fabian Davamani, Sumaiya Zabin Eusufzai, Farah Rashid, Nafij Bin Jamayet, Jue Ann Soh, Yen Yee Tan, Mohammad Khursheed Alam

**Affiliations:** 1 Division of Restorative Dentistry, School of Dentistry, International Medical University, Kuala Lumpur 57000, Malaysia; nafijjamayet@imu.edu.my (N.B.J.); yenyeetan@imu.edu.my (Y.Y.T.); 2 School of Medicine, International Medical University, Kuala Lumpur 57000, Malaysia; hareshkantilal@imu.edu.my; 3 Division of Clinical Oral Health Sciences, School of Dentistry, International Medical University, Kuala Lumpur 57000, Malaysia; suanphaik_khoo@imu.edu.my; 4 School of Health Sciences, International Medical University, Kuala Lumpur 57000, Malaysia; fabian_davamani@imu.edu.my; 5 School of Dental Sciences, Universiti Sains Malaysia, Kubang Kerian 16150, Malaysia; dr.sumaiya01@gmail.com (S.Z.E.); qazifarahrashid@gmail.com (F.R.); 6 LJ Lee Dental Clinic, Bandar Menjalara, Kuala Lumpur 52200, Malaysia; soh.jueann@student.imu.edu.my; 7 Department of Preventive Dentistry, College of Dentistry, Jouf University, Sakaka 72345, Saudi Arabia; mkalam@ju.edu.sa

**Keywords:** α-mangostin, *Garcinia mangostana*, antimicrobial

## Abstract

This systematic review aims to evaluate the antimicrobial activity of α-mangostin derived from *Garcinia mangostana* against different microbes. A literature search was performed using PubMed and Science Direct until March 2022. The research question was developed based on a PICO (Population, Intervention, Control and Outcomes) model. In this study, the population of interest was microbes, α-mangostin extracted from *Garcinia mangostana* was used as exposure while antibiotics were used as control, followed by the outcome which is determined by the antimicrobial activity of α-mangostin against studied microbes. Two reviewers independently performed the comprehensive literature search following the predetermined inclusion and exclusion criteria. A methodological quality assessment was carried out using a scoring protocol and the risk of bias in the studies was analyzed. Reward screening was performed among the selected articles to perform a meta-analysis based on the pre-determined criteria. Case groups where α-mangostin extracted from *Garcinia mangostana* was incorporated were compared to groups using different antibiotics or antiseptic agents (control) to evaluate their effectiveness. A total of 30 studies were included; they were heterogeneous in their study design and the risk of bias was moderate. The results showed a reduction in microbial counts after the incorporation of α-mangostin, which resulted in better disinfection and effectiveness against multiple microbes. Additionally, the meta-analysis result revealed no significant difference (*p* > 0.05) in their effectiveness when α-mangostin was compared to commercially available antibiotics. α-mangostin worked effectively against the tested microbes and was shown to have inhibitory effects on microbes with antibiotic resistance.

## 1. Introduction

**Figure 1:**
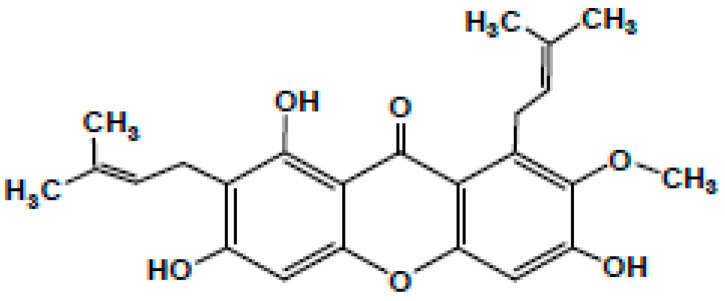
Chemical structure of a-mangostin.

*Garcinia mangostana* Linn. (commonly known as mangostin), family Guttiferae a delicious and aromatic fruit native to China, India, Indonesia, Malaysia, Myanmar, the Philippines, Thailand, and other parts of Southeast Asia, is synonymous with good health and has been labelled as a ‘super fruit’ [1,2]. The fruit is intense with a slightly acidic taste [3]. It is well established to have medicinal properties that make it an intrinsic part of traditional Chinese, Thai, and Ayurvedic medicines [4,5] These useful properties occur due to the bioactive compounds such as xanthones, particularly the α, β, γ-mangostins found in *Garcinia* fruits [6]. Besides these, tannins, terpenes, anthocyanins, benzophenones, depsidones, phloroglucinols, polyphenols, and flavonoids are also present in *Garcinia* fruits [3,4] Along with the fruits, the pericarp, hull, rind, peel, roots, and bark are also rich in mangostins [5]. These xanthones have a tricyclic aromatic structure, occur naturally in plants, and are antibacterial [7]. Of these, the most prominent is α-mangostin, which has been chemically identified as 3,6,8-trihydroxy-2-methoxy-1,7-bis (3-methyl but-2-enyl) xanthan-9-one as shown in Figure 1, with a molecular weight of 410.45 g/mol [8]. The only difference between α-mangostin and its β version is the presence of a methyl group instead of a hydroxyl group [9,10,11]. The pharmacological aspects of α-mangostin were reviewed and it has been reported that its bioactivities vary from acting as an antiseptic to being an analgesic, antipyretic, antimicrobial, anti-parasitic, anti-malarial, anti-leishmanial, anti-hypertensive, anti-obesity, anti-proliferative, antioxidant, anti-inflammatory, anti-aggregation of β-amyloid, anti-tumour, anti-allergic, anti-mutagen, anti-cancer, cholinesterase inhibition, anti-HIV, anti-cariogenic, pro-apoptotic, anti-ageing, and neuroprotective agent against Alzheimer’s and Parkinson’s disorders [12]. Thus, α-mangostin has been employed for its antimicrobial properties, especially for dental treatments [3,4,9,13,14,15,16]. This can be critical for dentists, as the primary recognized reason for failure in any endodontic treatment has been connected to the presence of microbes in the apical part of the root canal [17]. Recently, Kasemwattananaroj et al. (2019) reported its immunomodulatory properties for the lymphocyte lineage and cytokine generation in human peripheral blood mononuclear cells (PBMCs) [18].

The extent of the antimicrobial action of α-mangostin has been explored by many researchers and it has been observed that it is not just effective against bacteria, but also against other microbes such as fungi and mycobacteria. However, the level of antibacterial activity varies from species to species. The major species of bacteria that have been studied include facultative anaerobic Gram-positive species such as *Streptococcus*, *Enterococcus* [9,19], *Staphylococcus aureus* [9,14,19], *Propionibacterium acnes* [1,20,21], *Staphylococcus epidermidis* and *Salmonella* [1,21,22,23]. In addition to this, the germicidal action of α-mangostin was also successfully reported against Gram-negative bacteria such as *Pseudomonas aeruginosa*, *Klebsiella pneumoniae* [24], and *Escherichia coli* [8,14]; fungi such as *Candida albicans* [14] and *Aspergillus niger* [8]; mycobacteria such as *Mycobacterium tuberculosis*; and viruses such as dengue [25]. In addition to this, Charernsriwilaiwat et al. (2013) successfully loaded an electrospun chitosan nanofiber mat with α-mangostin and used it to help in wound healing processes [26]. The mode of action of the mat starts with the penetration and breakdown of the lipid membranes due to the strong hydrophobic bonds or CAMP-like molecules attached to the bacterial surface via electrostatic bonds [27,28]. In some cases, the isolated compound was modified to match the needs, as there was no observed activity with the Gram-negative bacteria [24].

Alternate sources of α-mangostin from plant species other than *G. mangostana* and their bioactivities have also been reported by several researchers. Negi et al. (2008) described the antibacterial properties of the fruit rinds of *G. cowa* and *G. pedunculata* against *Bacillus cereus*, *B. coagulans*, *Bacillus subtilis*, *Staphylococcus aureus*, and *E. coli* [29]. Taher et al. (2012) studied the chemistry of the stembark of *G. malaccensis* and observed its activity against *S. aureus* and *B. anthracis* [30]. As alternatives to these plant products, certain chemicals such as chlorhexidine digluconate, [31] alexidine, chlorhexidine, cetrimide [32], and sodium hypochlorite [33] have been employed as topical antimicrobial agents. However, these have been known to cause harmful side effects such as allergic reactions and irritation [34]. There are hardly any reviews consolidating the action of mangostins in overcoming these microbial infections. Moreover, Chen et al. (2018) pointed out that the present knowledge does not give a clear picture of the mechanism of action of α-mangostin [10]. Therefore, an in-depth study of the advantages of α-mangostin as a potent antimicrobial agent is imperative for the development of new forms of microbial agents. This systematic review aims to fill this lacuna in the use of plant products as antimicrobial agents using data from experimental studies. This systematic review will extend our knowledge of the therapeutic utilization of α-mangostin concerning the development of antimicrobial agents and the extent of their interactions. α-mangostin may be beneficial as an agent against specific infections in human beings as well as in other applications such as the development of germ-free food products and cosmetics.

## 2. Materials and Methods

### 2.1. Study Design and Selection

This is a systematic review of experiments that aimed to measure the antimicrobial activities of α-mangostin extracted from *Garcinia mangostana*. This systematic review was carried out per the Preferred Reporting Items for Systematic Review and Meta-Analysis (PRISMA) guidelines [35].

### 2.2. PICO

The population or patient or problem, intervention or exposure, comparison, or control (microbes), intervention or exposure and outcome (PICO) principal strategy used for the structured review questions was as follows:

1. Population/problem: microbes.
2. Intervention or exposure: use of α-mangostin extracted from *Garcinia mangostana*.
3. Comparison or control: antibiotics.
4. Outcome: antimicrobial activity of α-mangostin against studied microbes.

Based on these, the following free form of the research question was constructed:

“Does α-mangostin extracted from *Garcinia mangostana* have effective antimicrobial properties?”

### 2.3. Search Strategy

Systematic searches were performed using standard electronic databases such as PubMed and ScienceDirect. These databases were selected based on the researchers’ belief that these repositories offered articles of the highest quality and were most relevant to the topic of the research. In the PubMed database, the diagnostic search terms utilized were “*Garcinia mangostana*”, “mangostin”, and “anti-bacterial” or “anti-fungal” or “antimicrobial” or “anti-infective agents”. Each database was searched from inception until March 2022. Different keywords in different combinations were used alongside these two terms to conduct the searches. Boolean search operators (AND, OR) were used to connect keywords where appropriate. Quotation marks were used to search for an exact phrase, and the truncation symbol (*) was used where appropriate to search for all words starting with a particular combination of letters, such as *Garcinia mangostana*, mangostin, alpha mangostin, anti-bacterial agents, anti-infective agents, antimicrobial, etc. For Science Direct, search words such as *Garcinia mangostana*, mangostin, alpha mangostin, α-mangostin, antimicrobial, antibacterial, and antifungal were used. The search was complemented by the manual screening of the shortlisted papers and individually checked for eligibility criteria. Duplicate references were removed as and when they were detected by the researcher. An example of the search strategy that was used for PubMed is illustrated in Table 1.

### 2.4. Eligibility Criteria for Inclusion of Studies

#### 2.4.1. Inclusion Criteria

All the full-text articles were considered for assessment. Only studies where the source plant was *Garcinia mangostana* and the antimicrobial agent was in the chemical form of α-mangostin were included. Moreover, quantitative results were included, and the language was restricted to studies in English and full-text. The articles were screened according to their titles, plant sources, main constituents, and the name of the microbe to exclude irrelevant papers. However, no restrictions in terms of time were imposed for the inclusion of studies.

#### 2.4.2. Exclusion Criteria

All plant sources other than *Garcinia mangostana*, such as *G. cowa, G. malaccensis, G. smeathmanni*, and *Tetrigona melanoleuca* were excluded from the study. Additionally, the studies were screened for the mention of α-mangostin specifically. Any studies related to β- or γ- mangostins or any other xanthones that did not mention the specific type of mangostin or plant species used were also excluded from this study. Additionally, all review papers, abstracts, and book chapters were eliminated from this study. Moreover, all papers which had studied α-mangostin from *Garcinia mangostana,* but which did not mention any microbes were also removed from this study.

### 2.5. Data Extraction and Analysis

The characteristics of the included studies were extracted independently using a standardized form. These included the country of the study; the antimicrobial agent used; the plant part used in preparing α-mangostin; the types of microbes involved, and antibacterial activity measured in terms of minimum inhibitory concentration (MIC), minimum bactericidal concentration (MBC), or minimum fungicidal concentration (MFC).

#### Outcome Measures

The primary outcomes measured were the antimicrobial activity of α-mangostin against studied microbes.

### 2.6. Quality Assessment [Risk of Bias]

A critical quality assessment of the included studies was conducted using the Joanna Briggs Institute (JBI) critical appraisal (JBI, 2017) [36]. The nine questions are shown in Appendix A. One point was assigned to every ‘yes’ answer, while answers of ‘no’ or which were unclear were given zero points. Data extraction was carried out independently by two reviewers based on a set of items considered key to the study outcome. Any disagreements between the reviewers were resolved by reaching a consensus, and, when necessary, differences were resolved by a third reviewer. A quality assessment was used to categorize the level of evidence provided by the included studies.

### 2.7. Meta-Analysis Scoring Criteria Assessment by Using Newcastle–Ottawa

The meta-analysis was carried out based on articles that evaluated the effectiveness of α-mangostin derived from *Garcinia mangostana* as an antimicrobial agent (case group) and compared them with different commercially available antibiotics (control group). A modified Newcastle–Ottawa scoring scale was used to determine the eligibility criteria for this meta-analysis. Please refer to Table 2 for the scoring scale. Among all the articles, only those articles were selected that received a score of 3 and others that scored <3 were excluded from this meta-analysis. The excluded articles along with their scores are recorded in Table 3.

#### Data Extracted for Meta-Analysis

The meta-analysis result was synthesized using the statistical method of inverse variance of random effect with a 95% confidence interval. Cochrane Review Manager 5.4.1 was used to evaluate the heterogenicity with Tau^2^, chi^2^, *p*-value, and I^2^ and test for the overall effect with Z.

## 3. Results

### 3.1. Search Results

A total of 55 and 39 potentially relevant papers were identified using the shortlisted search words in the PubMed and ScienceDirect databases, respectively (Table 4). After reading the articles, manual screening was conducted, keeping the inclusion and exclusion criteria in mind. All the 94 articles included had been published as full texts. Of these, Samprasit et al., 2014, was the one paper not found as a full text in the library of the researcher [37]. The information required for screening was obtained from the abstract of that paper. Within the PubMed database, 6 out of 55 articles were excluded, as 2 of them were reviewed, 2 of them involved the use of *G. cowa* as the plant source, 1 of them involved *G. malaccensis* as the plant source, and 1 of them used *Tetrigona melanoleuca* as the plant source. *G. mangostana* was included in 49 articles. However, α-mangostin was used in only 33 articles. One of the articles mentioned γ-mangostin, and 15 of them failed to mention the type of mangostin or tested different derivatives/xanthones in their studies. Therefore, these 16 articles were excluded from our analysis. Three articles were later excluded as no microbes were involved. Therefore, 28 relevant articles on this subject were obtained from the PubMed database.

**Figure 2:**
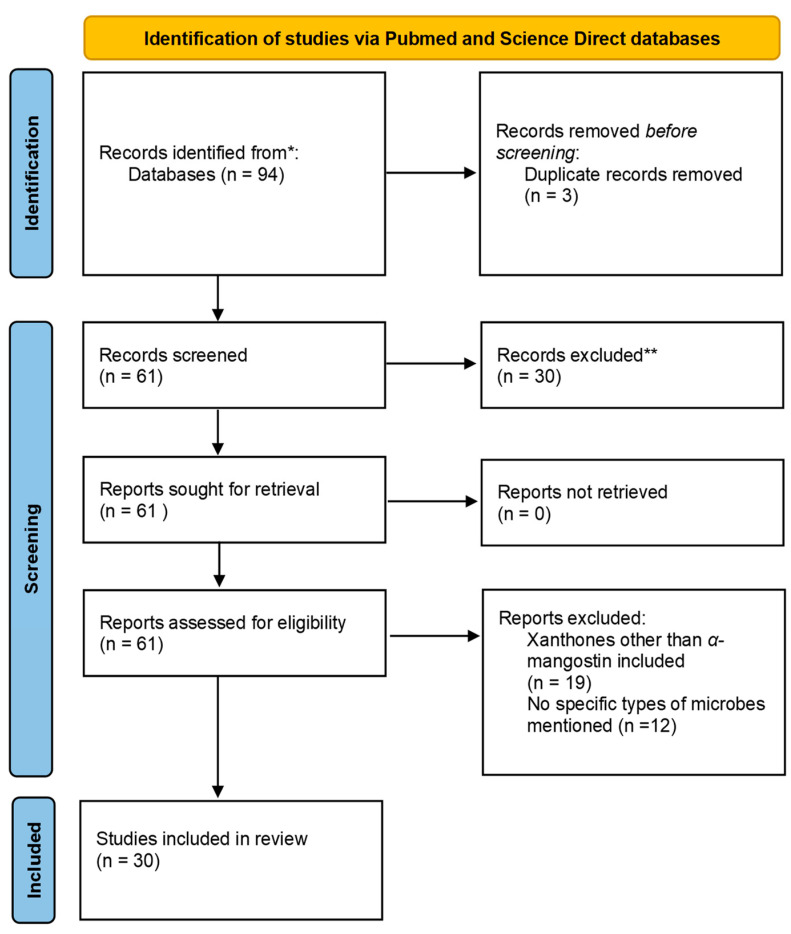
Flowchart demonstrating the PRISMA chart. * Records Identified; ** Records Excluded.

Similarly, 12 relevant articles were found from the list of articles obtained after screening the Science Direct database and removing the reviews. Of these, 9 involved studies on *G. mangostana*, and only 6 involved α-mangostin extracted from the fruit. Of these six articles, four were determined to be repeats from the PubMed search. Therefore, the Science Direct search gave us two unique articles to be included in this study. The PRISMA 2020 flowchart used is presented in Figure 2 [35].

### 3.2. Methodical Characterization of the Included Studies

Table 3 presents a summary of the 30 studies shortlisted for our review. These selected articles were published between the years 1996 and 2022. Many of them were reported from Thailand and Vietnam. However, there were a few instances from other countries such as Malaysia [38], Saudi Arabia [39], and Indonesia [7].

### 3.3. Risk of Bias in the Included Studies

All the included studies were evaluated for their risk of bias using the JBI appraisal tool (2017) as depicted in Table 5 [36]. The overall risk of bias in the included studies was moderate in all the cases. The differences in the studies were between the use of a control group and the use of some statistical tool for analysis. There was no control group in three studies [37,40,41], while statistical analysis was missing from 11 studies (Table 4). The quality of the score varied from five to six in total. The blinding of the participants was not possible due to the nature of the studies. Moreover, the allocation of the intervention groups was not concealed from the allocator in any of the studies. There were no multiple measurements of the outcome either before or after the intervention/exposure in any of them. Moreover, the reliability of the outcome was also unclear in all cases. The sixth question regarding the completion of the follow-up and any differences between groups in terms of whether their follow-up was adequately described and analyzed was not applicable for this study.

### 3.4. Meta-Analysis Result

Based on the predefined scoring criteria, eight articles were selected for this meta-analysis. These articles scored three and met all inclusion criteria. However, the rest of the articles that did not satisfy the criteria and scored <3, were excluded and are noted in Table 3.

**Figure 3:**
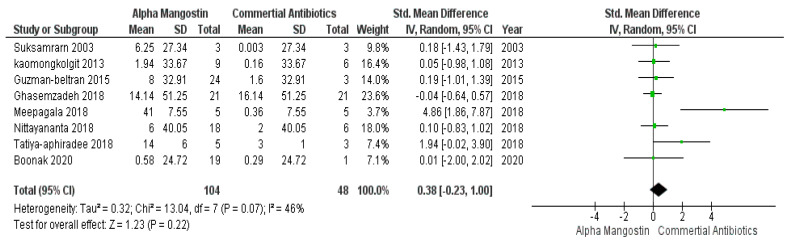
Forest plot of studies on commercially available antibiotics and alpha mangostin.

In Figure 3, studies those used commercially available antibiotics are plotted in control groups and those that incorporated α-mangostin are in the case group. This meta-analysis result evaluated that, based on the effectiveness, there were no significant differences (*p* > 0.05) between commercially available antibiotics and α-mangostin extracted from *Garcinia mangostana,* which means that both worked parallelly against different microbes. While the overall forest plot is showing no significant differences as the black diamond within Figure 3 exhibited inclination within the vertical line (i.e., the line of no effect), one study by Meepagala et al. [42] showed a significant difference hence the green square along with the horizontal line is situated away from the line of no effect that means individually the study showed an inclination towards the commercially available antibiotics over α-mangostin at a 95% confidence interval, I^2^ was 46% with heterogeneity Tau^2^ = 0.32, Chi^2^ = 13.04, df = 7 (*p* = 0.07), and test for overall effect, Z = 1.23 (*p* = 0.22).

## 4. Discussion

### 4.1. Standardization of the Garcinia Mangostana Extracts

The pericarp (hulls and peels) of *G. mangostana* was the most used plant part in the majority of studies included in this review; however, stem bark was used by Sakagami et al. (2005) [9]. The process of the extraction of xanthones and their isolation was initiated by the fractionation of the plant parts with hexane, benzene, acetone, and methanol; then, they were chromatographed on silica gel [7,9]. Methylene chloride was also used as a solvent and isolated β-mangostin along with α-mangostin, while Chomnawang et al. (2009) used chloroform and ethyl acetate in combination with other substances [43]. Pothitirat et al. (2009) extracted α-mangostin from *G. mangostana* using maceration and concentration techniques to avoid degradation and tested them for other bioactive compounds such as total phenolic compounds employing the Folin–Ciocalteu method, total flavonoids using the aluminum chloride colorimetric process, and total tannins using a protein precipitation assay [21]. The structural identification of the active isolates of α-mangostin was conducted using a spectrophotometer [7] or HPLC [1,21]. It was observed that the extracts from the younger fruits were richer in phenolics, flavonoids, and tannins compared to the older ones. To evaluate the actual effectiveness of α-mangostin against microbes, some studies had made comparisons between commercially available antibiotic agents and α-mangostin. These findings are in agreement with the current meta-analysis results which suggest no significant differences (*p* > 0.05) between the two groups.

### 4.2. The Efficacy of α-Mangostin on Microbes

From the data in Table 6, it can be summarised that xanthones—in particular, α-mangostin—are naturally-occurring antimicrobial agents. Iinuma et al., as early as 1996, reported the intense antibacterial properties of extracts obtained from *G. mangostana* and *G. dioica* against methicillin-resistant *Staphylococcus aureus* [7]. Similarly, Suksamrarn et al. (2003) has also reported that a-mangostin exhibited a strong inhibitory effect against *Mycobacterium Tuberculosis* when evaluated against standard drugs Rifampicin, Isoniazid, and Kanamycin [44]. Along with methicillin-resistant *S. aureus*, Sakagami et al. (2005) explored the possibility of employing antibiotics such as ampicillin, gentamicin, minocycline, and vancomycin hydrochloride in combination with α-mangostin against vancomycin-resistant *Enterococci* (VRE) with success; however, α-mangostin was found to be ineffective against *E. coli*, *Proteus vulgaris*, *Pseudomonas aeruginosa*, *Klebsiella pneumoniae*, and *Serratia marcescens* [9]. Chomnawang et al. (2009) evaluated extracts from 17 different medicinal plants, including *Garcinia*, against *S. aureus*. Moreover, in addition to *G. mangostana*, inhibition was observed for other extracts as well. However, maximum inhibition was observed for *G. mangostana*, followed by *Eupatorium odoratum* [43]. Pothitirat et al. (2009) observed that the extracts from the younger fruits were richer in phenolics, flavonoids, and tannins compared to those from older ones. However, the reverse was true for antibacterial properties, where the rinds of matured fruit showed an increased amount of α-mangostin, indicating their higher levels of bacterial inhibition [21]. Therefore, it can be suggested that the age of the fruit has an impact on the expected application of α-mangostin. For cosmetic purposes, younger fruits are better; however, for antimicrobial properties, matured ones will function better.

Pothitirat et al. (2010) tested the antimicrobial properties of α-mangostin extracted by different means against *Propionibacterium acnes* and *Staphylococcus epidermidis.* Among all five methods of extraction—maceration, percolation, magnetic stirring, ultrasonic apparatus, and the Soxhlet process—the more efficient antimicrobial properties were observed in extracts obtained through the Soxhlet method [1]. In another study by Nguyen and Marquis (2011), cariogenic oral bacteria such as *Streptococcus mutans* were tested against α-mangostin, and it was observed that the glycolysis, membrane-bound enzymes, glycolytic enzymes, alkali production, and respiration of the bacterial cells were inhibited by α-mangostin. Moreover, it had a killer effect on the bacteria at high concentrations [45]. Arunrattiyakorn et al. (2011) carried out the first microbial transformation of α-mangostin into four modified novel xanthones and tested them against endophytic fungi such as *Colletotrichum gloeosporioides*, *Neosartorya spathulate*, and *Mycobacterium tuberculosis.* Only two of the five derivatives were found to be effective [46]. Kaomongkolgit et al. (2013) explored the effect of α-mangostin on *Enterococcus faecalis*, a bacterium commonly found in root canal irrigations, and observed it to have anti-bactericidal properties [47]. Additionally, the current study also supports the claim because within the forest plot there are no significant differences when a comparison was made between α-mangostin and commercially available antibiotics. Moreover, α-mangostin had a higher rate of killing the microbe than chlorhexidine (CHX), which is commonly used as an irrigating solution in root canal procedures. Koh et al. (2013) investigated the antimicrobial properties of α-mangostin against the methicillin-resistant *Staphylococcus aureus* and reported it to be the most potent anti-infective agent. The mode of action by which α-mangostin controls the bacteria was also elaborated. It was reported that α-mangostin targets the membrane of bacteria by depolarizing it and inducing approximately 37% leakage due to vesicle lysis within a very short period (about 5 to 10 min) [28]. Based on pharmaceutical nanotechnology, alternate forms of healing using nanofiber mats as effective wound dressings were developed and *G. mangostana* was integrated into these through the ultra-spinning method [26]. The mats created were then tested against *S. aureus* and *E. coli* and found to be highly bactericidal and nontoxic and to possess high tensile strength. The synergistic effects of plant products and antibiotics on enhancing the ‘antimicrobial spectrum’ were evaluated by Seesom et al. (2013) [48]. Al-Massaarani et al. (2013) investigated the antimicrobial properties of α-mangostin against multiple microbes such as *Plasmodium falciparum*, *Leishmania infantum*, *Trypanosoma cruzi*, *T. brucei*, *Candida albicans*, *Escherichia coli*, *Pseudomonas aeruginosa*, *Bacillus subtilis*, *Staphylococcus aureus*, *Mycobacterium smegmatis*, *M. cheleneoi*, *M. xenopi*, and *M. intracellulare* and reported a moderate amount of bacterial selectivity, leading to inconclusive results regarding its antimicrobial action [39]. Nguyen et al. (2014) reported that the virulent aspects of *S. mutans* and its biofilms were curtailed by α-mangostin [40]. Similarly, Asasutjarit et al. (2014) also reported on the anti-bacterial properties of α-mangostin against *Propionibacterium acnes*, starting from amounts of 1 to 32 μg/mL of α-mangostin [49].

Mohamed et al. (2014) pointed out that α-mangostin had a quorum-sensing inhibitory action against *Chromobacterium violaceum*, a weak inhibitory activity against *E. coli* and *S. aureus*, and no action against *Candida albicans* [3]. In 2015, Samprasit et al. (2015) reported the use of *G. mangostana*-integrated mucoadhesive electrospun nanofiber mats against pathogens involved in dental caries. The antibacterial activity of these nanofiber mats was successfully tested against *S. mutans* and *S. sanguinis*. A nanofiber mat imbued with *Garcinia* extract showed the highest bactericidal activity and decreased both in vivo and in vitro oral bacteria [50]. The effect of α-mangostin in synergy with antibiotics such as oxacillin on oxacillin-resistant *S. saprophyticus* was studied by Phitaktim et al. (2016). It was observed that the growth of the bacterial strain was restricted by isolated α-mangostin in conjugation with the antibiotic. The 1,4-benopyrone structure of α-mangostin was responsible for restricting the bacteria. It was suggested that the mode of action for inhibiting the bacteria follows a three-step process. First, the cytoplasmic membrane is disrupted, causing increased permeability. This is followed by the restriction of the β-lactamase activity and, lastly, the impairment of the peptidoglycan [13]. In vivo antibacterial activity against methicillin-resistant *Staphylococcus aureus* was also reported by Tatiya-aphiradee et al. (2016) in the pericarp of *G. mangostana* using a superficial skin infection following the tape-stripping mouse model. This was the first study that evaluated the efficacy of the use of *Garcinia* extracts in infections in mice. It was also suggested that α-mangostin extract is an optimal candidate for the development of an alternate topical formulation to overcome infections [16]. The sensitivity of α-mangostin toward *Mycobacterium tuberculosis* was evaluated and compared against commercially available antibiotics by Guzmán-Beltrán et al. (2016). It was observed that α-mangostin was able to inhibit the infection at both high and low concentrations in human macrophages which means the study is in agreement with current study findings [51]. Narasimhan et al. (2017) assessed the use of α-mangostin and its synthetic derivative byproducts as antimicrobial agents against microbes contaminating food packaging, such as *E. coli*, *Bacillus subtilis*, *S. aureus*, and *P. aeruginosa*, and fungi such as *Candida albicans* and *Aspergillus niger.* The zone of inhibition was measured for each bacterium, and all 13 derivatives displayed antimicrobial properties against all the bacteria and fungi investigated [8]. Phuong et al. (2017) isolated α-mangostin from the peel of mangosteen fruit and examined its antibacterial activity against three strains of biofilms of *S. aureus* in Vietnam. It was proven that α-mangostin was the key antimicrobial compound. However, it was speculated that the α-mangostin also interacted with the extracellular matrix proteins along with the membrane proteins [23]. Ghasemzadeh et al. (2018) optimized the extraction process of α-mangostin from the plant source to improve the quality of the α-mangostin extracted and investigated it against various bacterial strains such as *Listeria ivanovii*, *Staphylococcus aureus*, *Mycobacterium smegmatis*, *Steptococcus uberis*, *Vibrio parahaemolyticus*, *Enterobacter cloacae*, and *E. coli*. There was a significant difference in the antimicrobial activity between the optimized and non-optimized extracts. The inhibition zone was the largest in the extracts optimized against *S. aureus*. This was reported to be the first study focusing on the antimicrobial activities of optimized extracts against microbes, apart from *S. aureus* and *E. coli* [38]. Nittayananta et al. (2018) extended the use of natural plant products such as α-mangostin, which is a common antimicrobial agent used against oral pathogens in oral sprays. α-mangostin was evaluated against *Candida albicans*, *Streptococcus mutans*, and *Porphyromonas gingivalis*, and it was observed that α-mangostin restricted the growth of these microbes without causing cytotoxicity. Therefore, it was suggested as a complementary mode of treatment along with conventional dental treatments [52]. Meepagala et al. (2018) discovered that α-mangostin was found to be 10-fold less active than γ-mangostin against catfish pathogens, *Flavobacterium columnare* [42]. In contrast to a study carried out by Tatiya-aphiradee et al. (2016) [16], it was revealed that MRSA infected wounds treated with *G. mangostana* Pericarp Extract (GME) were completely healed on the 10th day as compared to incomplete healing when treated with α-mangostin. Tatiya-aphiradee et al. (2019) suggested that the greater anti-inflammatory and anti-bacterial activity of GME is attributed to the presence of other constituents in the GM pericarp [53]. Recently, Chokpaisarn et al. (2019) reported the use of Ya-Samarn-Phlae, a type of traditional Thai medicine containing *G. mangostana*, along with *Curcuma longa*, *Oryza sativa,* and *Areca catechu* against *Pseudomonas aeruginosa.* It showed a high level of inhibition of the bacteria [54]. Larsuporm et al. (2019) performed the first in vitro study against *Staphylococcus pseudintermedius* isolated from cases of canine pyoderma, which are commonly seen in dogs and cats. The results revealed that α-mangostin in mangostin crude extract measured by HPLC was effective against two strains of *Staphylococcus pseudintermedius*, MRSP and MSSP with no significant difference reported between both. It was suggested by the authors that mangosteen crude extract might be a good substitute against chlorhexidine due to reports of chlorhexidine resistance in MRSA and increasing MICs among humans [41]. Five xanthones were isolated from *C. cochinchinense* and *G. mangostana* and tested for antibacterial activity against MRSA and *P. aeruginosa* as described by Boonak et al. (2020). One of the isolates, α-mangostin, exhibited the highest antibacterial activity; however, it possessed poor pharmacokinetic properties rendering it unsuitable to be used in the in-vivo model due to hepatoxicity and mutagenicity problems. Hence, α-mangostin analogues were produced by partially modifying the xanthone under the acidic condition which was then proven to show high anti-MRSA and *P. aerugionsa* activity along with better pharmacokinetic properties as tested by ADMET software. It is also noted that one of the analogues exhibited a synergistic effect against MRSA and *P. aeruginosa* when coupled with vancomycin [55]. Thus, our study was able to consolidate the bactericidal tendencies of α-mangostin against various forms of microbes, which may be beneficial for controlling infection in general. An antimicrobial study performed in Malaysia concluded that, without any combination with the other antimicrobial agent, α-mangostin cannot play the role of an effective antimicrobial agent whereas commercial antibiotics alone could show effective results in fighting against microbes [56]. These findings are partially contrary to the findings of the current study but are supported by Meepagala et al., who suggested the commercially available antibiotics show a better antimicrobial effect over α-mangostin [42].

Another study proved that α-mangostin is not effective in the inhibition of gram-negative bacteria. However, α-mangostin alone or in a mixture with gentamicin against vancomycin-resistant *Enterococci,* and α-mangostin in a mixture with vancomycin hydrochloride against methicillin-resistant *S. aureus,* could be more effective in infection control measures. α-mangostin could thus play a role, used alone or in combination, in the inhibition of certain bacteria, as is partially supported by our study results. However, it is suggested that α-mangosteen should use in a combination with any commercial antibiotic agent to maximize its antimicrobial property [9]. It was also discovered from our study that certain microbes, such as the bacteria *Enterococcus faecalis* and *Staphylococcus epidermidis* and fungi *Candida albicans*, cause periodontic infections that have rarely been studied by researchers. This research can form the basis for future studies in many disciplines of medicine and dentistry.

### 4.3. Strengths and Limitations of This Study

The major strengths of this review involve the lack of any time limitations and the inclusion of all studies in which the fruits of *Garcinia mangostana* were used as a direct plant source. Moreover, this review compiles information from the major studies on this topic conducted across the world and gives a brief list of all the microbes that have been tested against α-mangostin. Our study also consolidates the amount of inhibition caused by these substances along with the antibiotic-resistant species. However, no study is without its limitations. Our study was subject to all limitations experienced by systematic reviews. Moreover, this review was limited to only two database searches and was confined to experimental studies concerning α-mangostin extracted from the plant parts of *Garcinia mangostana.* Other forms of mangostins or other plant sources, despite having microbiocidal properties, were not considered. No strict guidelines regarding the comparison with the control were followed. The reports were mostly confined to Southeast Asia. The assessment of study quality was limited due to the experimental nature of the studies. Moreover, the methodologies used by the reported studies were not of a standardized nature.

## 5. Conclusions

From the results of our systematic review and meta-analysis, it can be concluded that α-mangostin is effective against multiple microbes, including ones with antibiotic resistance. Additionally, α-mangostin, though plant-based, produced similar antimicrobial activity in comparison with commercially available antibiotics. To the best of our knowledge, this is the first systematic review on the potential of α-mangostin as an antimicrobial agent. Our study results suggested that both α-mangostin and commercial antibiotics showed similar antimicrobial effects in the inhibition of reported microorganisms such as *M. tuberculosis*, *E. faecalis*, *L. ivanovii*, *S. aureus*, *M. smegmatis*, *S. uberis*, *V. parahaemolyticus*, *E. cloacae*, *E. coli*, *F. columnare*, methicillin-resistant *S. aureus*, *C. albicans*, *S. mutans*, *P. gingivalis*, *S. typhi*, *S. sonei* and *P. aeruginosa*.

### Implications for Future Studies

The lack of natural healing agents in today’s world necessitates the investigation of many pharmacological aspects of natural antimicrobial agents such as α-mangostin. The biggest advantage of α-mangostin lies in its non-toxic and safe nature. Moreover, the use of antibiotics as antimicrobial agents must also consider any toxic side effects. Thus, this study was undertaken in an attempt to understand the functioning of α-mangostin from *Garcinia mangostana* as an effective antimicrobial agent. Compounds such as this are the backbone of the drug industry and have wide pharmaceutical applications in the field of medical science. It was found that α-mangostin plays an active role in wound healing and is also useful in cosmetology and the food packaging and processing industry. Our study’s scope includes the usefulness of this substance to researchers from microbiology, botany, and ethnopharmacology; drug therapists; and all kinds of doctors dealing with the control of infection in various body parts. Based on our study, it can be suggested that α-mangostin is an ideal candidate for the development of a new category of antimicrobial compounds to overcome antibiotic resistance; these compounds have potential applications in the area of future pharmacology and healthcare and may also be used in the treatment of immune diseases. Apart from this, these compounds can be advantageous in the treatment of dental issues and for wound-healing purposes. The range of uses of α-mangostin can be vast, extending from the field of biomedicine to biomaterials, biomedical devices, food preservation, and many others.

**Table 6:** Characteristics of the studies and their outcomes and results.

Study ID	Author	Country of Study	Main Antimicrobial Agent	Plant Part Used	Control	Microbes	Method	MIC * of Test		MIC * of Control		MBC **/MFC ***	
								Inhibitory (mm)	Count (µg/mL)	Inhibitory (mm)	Count (µg/mL)	Test Count (µg/mL)	Control Count (µg/mL)
1.	Chokpaisam et al. (2019) [54]	Thailand	Ya-Samarn-Phlae (YSP)	Pericarp of *Garcinia*, seeds of *Areca catechu* and *Oryza sativa* and rhizome of *Cucurma longa*	PBS	*P. aeruginosa*	Crystal violet assay	12.29 μm	-	18	-		
2.	Ghasemzadeh et al. (2018) [38]	Malaysia	α-mangostin	Pericarp	Ciprofloxacin	*L. ivanovii*, *S. aureus*, *Mycobacterium smegmatis*, *Streptococcus uberis*, *Vibrio parahaemolyticus*, *Enterobacter cloacae*, *E. coli*	DPPH assay, FRAP assay,	17, 18, 16, 14, 12, 12, 10		18, 16, 17, 18, 14, 12, 12, 10			
3.	Narasimhan et al. (2017) [8]	-	α-mangostin and synthetic derivatives	Dried fruits	Ciprofloxacin	*E. coli*, *Bacillus subtilis*, *S. aureus*, and *P. aeruginosa*,	Muller Hilton agar plates	4	50	11	50		
					Ketoconazole	*Candida albicans*, *Aspergillus niger*	Disc diffusion method	13	100				
4.	Phuong et al. (2017) [23]	Vietnam	α-Mangostin	Peels	PBS	*Staphylococcus aureus*	Membrane activity assay		4.58–9.15 μmol/L			2 folds higher	
5.	Phitaktim et al. (2016) [13]	Thailand	α-mangostin alone and combination with oxacillin and nisin	Matured dried fruit hulls	*S. aureus*	*S. saprophyticus*	MTT assays		8		4		
6.	Tatiya-Aphiradee et al. (2016) [16]	Thailand	α- and γ-mangostin	Crude dried pericarp	Gentamicin, Erythromycin	Methicillin-resistant *Staphylococcus aureus*	Agar well diffusion assay	10	0	6.25	>10,000	100	>10,000
7.	Samprasit et al. (2015) [50]	Thailand	α-mangostin	Pericarp	0.2% *w*/*v* chlorhexidine	*S. mutans* and *S. sanguinis*	MTT assay		0.1 mg/mL		1 mg/mL		0.2 mg/mL
8.	Nguyen et al. (2014) [40]	Vietnam	α-mangostin	Peels		*S. mutans*	F-ATPase and phosphotransferase system (PTS) assays						
9.	Mohamed et al. (2014) [3]	Vietnam	Mangostanaxanthones I and II, 9-hydroxycalabaxanthone, parvifolixanthone C, α-mangostin and rubraxanthone	Air-dried pericarps	Ampicillin	*Staphylococcus aureus*, *Bacillus cereus*, *Escherichia coli*, *and* *C. violaceum*	Agar plate diffusion and dilution	2	250	24	-		
					Fluconazole	*C. albicans* and *A. fumigatus*		0		20		NA	NA
10.	Asasutjarit et al. (2014) [49]	Thailand	α-mangostin	Air-dried rind	Amoxicillin	*Propionibacterium acnes*	Microdilution assay		0.5		0		
11.	Al-Massarani et al. (2013) [39]	Saudi Arabia	α-Mangostin	Pericarp	Amoxifen for MRC-5, chloroquine for *P. falciparum*, miltefosine for *L. infantum*, benznidazole for *T. cruzi* and suramin for *T. brucei.*	*C. albicans*, *Plasmodium falciparum*, *Leishmania infantum* and *Trypanosoma cruzi* and *T. brucei*, *Escherichia coli*, *Pseudomonas aeruginosa*, *Bacillius subtilis*, *Staphylococcus aureus*, *Mycobacterium smegmatis*, *M. cheleneoi*, *M. xenopi* and *M. intracellulare.*	Broth microdilution	NA	>200 µg/mL	NA	NA	NA	NA
12.	Seesom et al. (2013) [48]	Thailand	α-, ϒ-Mangostin	Pericarp	Penicillin	*Leptospira biflexa*	Broth microdilution method		200 to >800		0.39 to 6.25		
13.	Charernsriwilaiwat et al. (2013) [26]	-	α-Mangostin	Fruit hull	Penicillin	*Staphylococcus aureus* and *Escherichia coli*	Metal ion chelating assay		0.5			0.5	
14.	Koh et al. (2013) [28]	-	1,5,8-trihydroxy-3-methoxy-2-(3- methyl-2-butenyl) xanthone, γ-mangostin, garcinia E, α-mangostin and mangostenoneD	Fruit hull	Vancomycin	Methicillin-resistant *Staphylococcus aureus* (MRSA)	SYTOX green assay		3.125		0.78–1.56		
15.	Arunrattiyakorn et al. (2011) [46]	-	a-Mangostin (1), mangostin 3-sulfate (2), mangosteen 6-sulfate (3), 17,18-dihydroxymangostanin 6-sulfate (4) and isomangostanin 3-sulfate (5).	Fruit hull	Rifampicin, streptomycin, isoniazid, and ofloxacin	*Colletotrichum gloeosporioides* and *Neosartorya spathulata*	Green fluorescent protein microplate assay (GFPMA)	15.24 and 6.75 μm for 1 and 2, respectively, 3–5 showed no activity (MIC > 50 lg/mL).		0.36 × 10^−2^, 1.46 × 10^−2^, 0.29–0.54, 0.17–0.34, and 1.08–2.16 μm for rifampicin, streptomycin, isoniazid, and ofloxacin, respectively.			
16.	Nguyen et al. (2011) [45]	Vietnam	α-Mangostin	Peel	α-Mangostin with 25% ethanol	*Streptococcus mutans*	F-ATPase and phosphotransferase system (PTS) assays	70%					
17.	Pothitirat et al. (2010) [1]	Thailand	α-mangostin	Rind	other extracts (Hex, EtOH, H_2_O)	*S. epidermidis*, *P. acne*	Microdilution assay	15.63, 7.8–15.63	3.91 µg/mL	NA	7.81–500 µg/mL	15.63 µg/mL	31.25–(>500) µg/mL
18.	Pothitirat et al. (2009) [21]	Thailand	α-mangostin	Matured rinds	Pure α-mangostin	*S. epidermidis*, *P. acnes*	Microdilution assay	NA	15.63 for both	NA	1.95 (*P. acnes*), 3.91 (*S. epidermidis*)	15.63 (*P.acnes*), 31.25 (*S. epidermidis)*	1.95 (*P. acnes*), 3.91 (*S. epidermidis*)
				Young rinds	Pure α-mangostin	*S. epidermidis*, *P. acnes*	Microdilution assay	NA	15.63 (*P.acnes*), 31.25 (*S. epidermidis*)	NA		31.25 (*P.acnes*), 62.50 (*S. epidermidis*)	
19.	Chomnawang et al. (2009) [43]	Thailand	α-mangostin	Not mentioned	16 other medicinal plants	Methicillin-resistant *Staphylococcus aureus*, *S. epidermidis*	Disc diffusion and microdilution assay	11.3 ± 0.60 (*S. aureus*), 10.50 ± 0.70 (*S. epidermidis*) mm	0.039 mg/mL for all	Not detected to 19.70 ± 0.60 mm	0.625–(>5) mg/mL	0.156 for both (mg/mL)	≥5 mg/mL
20.	Sakagami et al. (2005) [9]	-	α-, β- Mangostin	Stem bark	Gentamicin	Vancomycin-resistant *Enterococci* (VRE)	Agar Dilution	NA	3.13 (α-Mangostin), 25 (β- Mangostin	NA	>100	NA	NA
						Methicillin-resistant *Staphylococcus aureus* (MRSA)	Agar Dilution	NA	6.25 (α-Mangostin), >100 (β- Mangostin	NA	>100	NA	NA
21.	Iinuma et al. (1996) [7]	Indonesia	α-Mangostin	Dried and ground pericarp	Vancomycin, Gentamycin	*Staphylococcus aureus*			1.57–>12.5		0.8 (Vancomycin) and 1.57 (Gentamicin)		
						*E. coli*	Bioassay		25		>25 (Vancomycin) and 25 (Gentamicin)		
22.	Guzmán-Beltrán et al. (2015) [51]	-	α-Mangostin, NDGA	-	Rifampicin at 0.4 μg/mL	*Mycobacterium tuberculosis*	Colourimetric assay		250 (NDGA), 62.5 (α-Mangostin)				
23.	Kaomongkolgit et al. (2013) [47]	Thailand	α-Mangostin	Dried pericarps	NaOCl and CHX	*Enterococcus faecalis*	MTT assay		1.97		0.15% (NaOCl), 2.5 (CHX)	3.94	0.31% (NaOCl), 5 (CHX)
24.	Nittayananta et al. (2018) [52]	Thailand	α-Mangostin and/or lawsone methyl ether (2-methoxy-1,4-naphthoquinone) (LME)	Pericarp	Gentamicin	*Candida albicans*	Microdilution assay		625 mg/mL			0.625 mg/mL	
						*Streptococcus mutans*	Microdilution assay		0.3125 mg/mL			>2.5 mg/mL	
						*Porphyromonas gingivalis*	Microdilution assay		2.5 mg/mL			2.5 mg/mL	
25	Meepagala et al. (2018) [42]	USA	α-mangostinγ-Mangostin(-)-Epicatechin	Pericarp of *Garcinia mangostana*	Florfenicol	*Flavobacterium columnare*	MTT assay ALM-00-173	-	41.0	-	0.36	-	-
26	Larsuprom et al. (2019) [41]	Thailand	a-mangostin	Pericarp of *Garcinia mangostana*		Methicillin-susceptible *S. pseudintermedius* (MSSP) methicillin-resistant *S. pseudintermedius* (MRSP)	Broth Microdilution Method	-	0.53 ± 0.35 µg/mL, 0.47 ± 0.27 µg/mL	-	-	-	-
27	Boonnak et al. (2020) [55]	Thailand	a-mangostin and derivatives norathyriol γ-Mangostin and deririatives dulxisxantone β-mangostin	CH2Cl2 extracts of the *C. cochichinense* resin *and G. mangostana* hulls	Vancomycin	MRSA *B. subtilis* *E. faecalis* VRE *S. typhi* *S. sonei* *P. aeruginosa*	Not stated	-	2.34 2.34 150 150 18.75 150 2.34		2.34	-	-
28.	Suksamsarn et al. (2003) [44]	Thailand	(1)a-mangostin	Fruit hulls and the edible arils and seeds of *Garcinia mangostana*	Rifam picin Isoniazid Kanamycin	*Mycobacterium tuberculosis*	Microplate Alamar Blue Assay	-	6.25	-	0.003–0.0047, 0.025–0.05 1.25–2.5	-	-
29.	Tatiya-aphiradee et al. (2019) [53]	Thailand	a-mangostin	Pericarp extract of *Garcinia mangostana*	Oxacillin Erythromycin	MSSA ATCC 9144 MSSA ATCC 23235 MRSA DMST4738 MRSA DMST20651 MRSA DMST20654	Micro-dilution method.	-	3.625 7.250 12.50 6.25 6.25	-	0.800 0.800 O—25.00 E—>400 >400 O—200 E > 400	-	-
30.	Samprasit et al. (2014) [37]	-	α-Mangostin	-		Oral microbes	Time kill assay	Only abstract available to the researcher					

* MIC = minimum inhibitory concentration; ** MBC = minimum bactericidal concentration; *** MFC = minimum fungal concentration.

## Figures and Tables

**Table 1:** Keywords used for the search strategy in PubMed.

Search Words	Results
“garcinia”	1898
“garcinia mangostana” OR (“garcinia” AND “mangostana”)	540
(“garcinia mangostana” OR (“garcinia” AND “mangostana”) OR “garcinia mangostana”) AND (“mangostin” OR “mangosteen”)	431
(“garcinia mangostana” OR (“garcinia” AND “mangostana” OR “garcinia mangostana”) AND (“mangostin”) OR “mangosteen”) AND Anti)	199
((((garcinia mangostana) OR garcinia)) AND ((((((mangostana) OR garcinia mangostana) OR mangosteen) OR mangostin) OR mangostin) OR alpha mangostin)) AND (((anti-bacterial agents) OR anti-bacterial agents OR anti-bacterial)) AND ((((((agents) OR anti-bacterial agents) OR antibacterial) OR anti-infective agents) OR anti-infective agents OR anti-infective)) AND (((agents) OR anti-infective agents) OR antimicrobial)	55

**Table 2:** Newcastle–Ottawa scoring criteria used for meta-analysis.

Score	Criteria
0	Inadequate description of case and control group Studies that did not clearly define their sample sizes along with their means and standard deviations
1	Studies that used another antimicrobial agent except for antibiotics and antiseptics as the control group Studies that used α-mangostin as an antimicrobial agent in both the case and control group
2	Studies that did not include α-mangostin as a major antimicrobial agent Studies that used fungi and parasites as micro-organisms
3	Studies included in this meta-analysis: Studies that provided a proper description of the case and control group Studies that clearly defined their sample sizes along with the mean and standard deviations Studies that incorporated α-mangostin in their case groups and compared it against different antibiotics and antiseptic agents

**Table 3:** Articles excluded from this meta-analysis with their reasons for exclusion.

Author Name (Year)	Title	Reason of Exclusion
Chokpaisam (2019)	Effects of a traditional Thai polyherbal medicine ‘Ya-Samarn-Phlae’ as a natural anti-biofilm agent against *Pseudomonas aeruginosa*	2
Larsuprom (2019)	In vitro antibacterial activity of mangosteen (*Garcinia mangostana* Linn.) crude extract against *Staphylococcus pseudintermedius* isolates from canine pyoderma	0
Larsuprom (2019)	In vitro antibacterial activity of mangosteen (*Garcinia mangostana* Linn.) crude extract against *Staphylococcus pseudintermedius* isolates from canine pyoderma	0
Narasimhan (2017)	Anti-bacterial and anti-fungal activity of xanthones obtained via semi-synthetic modification of α-mangostin from *Garcinia mangostana*	1
Nguyen (2017)	Antibiofilm activity of a-mangostin extracted from *Garcinia mangostana* L. against *Staphylococcus aureus*	1
Phitaktim (2016)	Synergism and the mechanism of action of the combination of α-mangostin isolated from *Garcinia mangostana* L. and oxacillin against an oxacillin-resistant *Staphylococcus saprophyticus*	1
Tatiya-Aphiradee (2016)	In vivo antibacterial activity of *Garcinia mangostana* pericarp extract against methicillin-resistant *Staphylococcus aureus* in a mouse superficial skin infection model	1
Mohamed (2014)	Mangostanaxanthones I and II, new xanthones from the pericarp of *Garcinia mangostana*	0
Asasutjarit (2014)	Physicochemical properties and anti-propionibacterium acnes activity of film-forming solutions containing alpha-mangostin-rich extract	0
Nguen (2014)	a-Mangostin disrupts the development of *Streptococcus mutans* biofilms and facilitates its mechanical removal	1
Samprasit (2014)	Mucoadhesive electrospun chitosan-based nanofiber mats for dental caries prevention	0
Samprasit (2014)	Antibacterial activity of *Garcinia mangostana* extracts on oral pathogens	0
Koh (2013)	Rapid bactericidal action of alpha-mangostin against MRSA as an outcome of membrane targeting	1
Al-Massarani (2013)	Phytochemical, antimicrobial, and antiprotozoal evaluation of *Garcinia mangostana* pericarp and α-mangostin, it is a major xanthone derivative	2
Charernsriwilaiwat (2013)	Electrospun chitosan-based nanofiber mats loaded with *Garcinia mangostana* extracts	0
Seesom (2013)	Antileptospiral activity of xanthones from *Garcinia mangostana* and synergy of gamma-mangostin with penicillin G	2
Arunrattiyakorn (2011)	Microbial metabolism of a-mangostin isolated from *Garcinia mangostana* L.	2
Nguyen (2011)	Antimicrobial actions of a-mangostin against oral *Streptococci*	0
Pothitirat (2010)	Free radical scavenging and anti-acne activities of mangosteen fruit rind extracts prepared by different extraction methods	0
Pothitirat (2009)	Comparison of bioactive compounds content, free radical scavenging, and anti-acne inducing bacteria activities of extracts from the mangosteen fruit rind at two stages of maturity	0
Chomnawang (2009)	Antibacterial activity of Thai medicinal plants against methicillin-resistant *Staphylococcus aureus*	1
Sakagami (2005)	Antibacterial activity of a-mangostin against vancomycin-resistant *Enterococci* (VRE) and synergism with antibiotics	1
Iinuma (1996)	Antibacterial Activity of Xanthones from Guttiferaeous Plants against Methicillin-resistant *Staphylococcus aureus*	1

**Table 4:** Summary of the screening process.

Database	Initial Hits	After Screening 1 (Only Research Articles Included)	After Screening 2 (Only *G. mangostana* Included)	After Screening 3 (Only α-Mangostin Included)	After Screening 4 (Type of Microbe Mentioned) Unique Records
PUBMED	55	53	49	33	28
Science Direct	39	12	12	9	2
Total	94	65	61	42	30

**Table 5:** Risk of bias of included studies.

	Author of the Study	Q1	Q2	Q3	Q4	Q5	Q6	Q7	Q8	Q9	Total
1	Chokpaisam et al. (2019)	1	1	1	1	0	na	1	unclear	1	6
2	Ghasemzadeh et al. (2018)	1	1	1	1	0	na	1	unclear	1	6
3	Narasimhan et al. (2017)	1	1	1	1	0	na	1	unclear	0	5
4	Phitaktim et al. (2016)	1	1	1	1	0	na	1	unclear	1	6
5	Tatiya-Aphiradee et al. (2016)	1	1	1	1	0	na	1	unclear	1	6
6	Samprasit et al. (2015)	1	1	1	1	0	na	1	unclear	1	6
7	Nguyen et al. (2014)	1	1	1	0	0	na	1	unclear	1	5
8	Mohamed et al. (2014)	1	1	1	1	0	na	1	unclear	0	5
9	Asasutjarit et al. (2014)	1	1	1	1	0	na	1	unclear	1	6
10	Al-Massarani et al. (2013)	1	1	1	1	0	na	1	unclear	0	5
11	Seesom et al. (2013)	1	1	1	1	0	na	1	unclear	0	5
12	Charernsriwilaiwat et al. (2013)	1	1	1	1	0	na	1	unclear	1	6
13	Koh et al. (2013)	1	1	1	1	0	na	1	unclear	0	5
14	Arunrattiyakorn et al. (2011)	1	1	1	1	0	na	1	unclear	0	5
15	Nguyen et al. (2011)	1	1	1	1	0	na	1	unclear	0	5
16	Pothitirat et al. (2010)	1	1	1	1	0	na	1	unclear	1	6
17	Pothitirat et al. (2009)	1	1	1	1	0	na	1	unclear	1	6
18	Chomnawang et al. (2009)	1	1	1	1	0	na	1	unclear	0	5
19	Sakagami et al. (2005)	1	1	1	1	0	na	1	unclear	0	5
20	Iinuma et al. (1996)	1	1	1	1	0	na	1	unclear	0	5
21	Phuong et al. (2017)	1	1	1	1	0	na	1	unclear	1	6
22	Guzmán-Beltrán et al. (2016)	1	1	1	1	0	na	1	unclear	1	6
23	Kaomongkolgit et al. (2013)	1	1	1	1	0	na	1	unclear	1	6
24	Nittayananta et al. (2018)	1	1	1	1	0	na	1	unclear	0	5
25	Meepagala et al. (2018)	1	1	1	1	0	na	1	unclear	1	6
26	Larsuprom et al. (2019)	1	1	1	0	0	na	1	unclear	1	5
27	Boonnak et al. (2020)	1	1	1	1	0	na	1	unclear	1	6
28	Suksamsarn et al. (2003)	1	1	1	1	0	na	1	unclear	1	6
29	Tatiya-aphiradee et al. (2019)	1	1	1	1	0	na	1	unclear	1	6
30	Samprasit et al. (2014)	1	1	1	0	0	na	1	unclear	1	5

No or unclear: 0 point. Yes: 1-2-3 points for low methodologic quality; 4-5-6 points for moderate methodologic quality; 7-8-9 points for high methodologic quality. na = not applicable.

## Data Availability

The data supporting this systematic review and meta-analysis are sourced from previously reported studies and datasets that have been cited. The processed data are available from the corresponding author upon request.

## Appendix A

Reviewer ______________________________________ Date_______________________________

Author_______________________________________ Year_________ Record Number_________

	Yes	No	Unclear	Not applicable
1. Is it clear in the study what is the ‘cause’ and what is the ‘effect’ (i.e., there is no confusion about which variable comes first)?	□	□	□	□
2. Were the participants included in any comparisons similar?	□	□	□	□
3. Were the participants included in any comparisons receiving similar treatment/care, other than the exposure or intervention of interest?	□	□	□	□
4. Was there a control group?	□	□	□	□
5. Were there multiple measurements of the outcome both pre and post the intervention/exposure?	□	□	□	□
6. Was follow up complete and if not, were differences between groups in terms of their follow up adequately described and analyzed?	□	□	□	□
7. Were the outcomes of participants included in any comparisons measured in the same way?	□	□	□	□
8. Were outcomes measured in a reliable way?	□	□	□	□
9. Was appropriate statistical analysis used?	□	□	□	□

Overall appraisal: Include □ Exclude □ Seek further info □

Comments (Including reason for exclusion)

______________________________________________________________________________________________

______________________________________________________________________________________________

____________________________________________________________________
